# Smart Materials: Cementitious Mortars and PCM Mechanical and Thermal Characterization

**DOI:** 10.3390/ma14154163

**Published:** 2021-07-27

**Authors:** Federico Orsini, Paola Marrone, Silvia Santini, Lorena Sguerri, Francesco Asdrubali, Giorgio Baldinelli, Francesco Bianchi, Andrea Presciutti

**Affiliations:** 1 Department of Architecture, Roma Tre University, 00153 Rome, Italy; federico.orsini@uniroma3.it (F.O.); paola.marrone@uniroma3.it (P.M.); lorena.sguerri@uniroma3.it (L.S.); 2 Department of Engineering, Roma Tre University, 00153 Rome, Italy; francesco.asdrubali@uniroma3.it; 3 Department of Engineering, University of Perugia, 06100 Perugia, Italy; giorgio.baldinelli@unipg.it; 4 Department of Physics and Geology, University of Perugia, 06100 Perugia, Italy; francesco.bianchi@unipg.it; 5 Department of Economy, Universitas Mercatorum, 00153 Rome, Italy; andrea.presciutti@unimercatorum.it

**Keywords:** PCM, smart materials, mechanical characterization, thermal characterization

## Abstract

Climate change (CC) is predominantly connected to greenhouse gas (GHG) emissions from the construction sector. It is clear how it is necessary to rethink construction materials in order to reduce GHG emissions. Among the various strategies proposed, recent research has investigated the potential of smart materials. This study in particular aims to develop an innovative building component that combines high energy performance with reduced thickness and weight. For this reason, the potential of Phase Change Materials (PCM) in cement-based mixes is investigated, comparing the performance of a traditional mix with two innovative mixes made with the addition of 3% and 7% PCM. This work characterizes the new material, analyzing its mechanical and thermal performance, highlighting how the mix strength decreases as the PCM ratio increases; however, both mixes may be considered suitable for masonry structures and may be classified as M5 and M15. Furthermore, from the analysis of the thermal performance, it emerges that the mix presents good behavior in terms of insulating properties.

## 1. Introduction

Favoring climate change (CC), greenhouse gases (GHGs), mainly produced by anthropogenic activities, are currently considered responsible for the increase in the global average temperature, which has risen by about +1 °C compared to the preindustrial era [1]. Due to GHGs, today, large, densely populated areas are becoming inhospitable [2].

The evidence of climate change and the serious consequences associated with it require a radical rethinking of urban settlements, buildings and construction materials. In this context, the need to develop sustainable buildings with reduced GHG emissions appears a necessary goal [3]. Favoring the use of renewable resources [4], adapting economic development to principles of the circular economy [5,6,7,8], producing low-carbon materials [9,10] and reducing energy consumption [11] through innovative high-performance materials and low-carbon emissions are some possible strategies that can be used to achieve this goal. Among these, the reduction of energy consumption in buildings is a fundamental strategy, considering that buildings are responsible for over 40% of GHG emissions. This reduction can be achieved by working, in addition to integrated renewable energy production systems, on the energy performance of the building envelope [12,13]; improvement of energy performance which goes from increasing the thermal performance of the envelope [14,15] both in terms of reducing heat loss and in terms of increasing the thermal lag. To try to achieve this goal, innovative superinsulating materials [16] or innovative super-insulating materials [16] or innovative smart materials have recently been developed, capable of adapting and interacting with the external environment, improving the performance of the envelope. Among the most recent smart materials, we find Phase Change Materials (PCMs). These materials can be defined as “smart” precisely because they perform and change their characteristics as the external environmental conditions change. This feature makes them interesting materials for construction to be used independently or hybridized with traditional materials, thus creating innovative high-performance composted materials. On the one hand, in fact, superinsulating materials reduce the thermal transmittance of the envelope, and on the other hand, PCMs allow increasing its thermal storage capacity. This latter aspect makes PCMs of particular interest and the subject of recent studies [17,18].

Several studies have analyzed PCM applications in the building construction sector. Desai et al. hybridized PCMs with construction materials and produced an “engineered” cement, observing how a 3% increase in PCM increases the specific heat capacity of the phase change temperature by 40% while maintaining compression strength at 28 MPa [19]. Cabeza et al. underlined how the addition of 5% PCM at 26 °C (110 kJ/kg) guarantees a compressive strength of over 25 MPa and tensile strength of over 6 MPa, which are valid results for structural use, and at the same time, reduces the internal temperature peaks by 1–2 °C, delaying them by 2 h [20]. Similar studies have also been conducted on cement-based mortars [21,22] compared with traditional mortars [23] or integrated with particles from recycled tires [24]. Sen et al. present a physics-guided multi-objective optimization procedure in order to develop mixture design of functional cementitious materials containing microencapsulated phase change materials, underling how this method can be used to assist the design of a variety types of functional cementitious composite and concrete [25]. PCMs have also been integrated into aluminum honeycomb panels (8 mm cell thickness, with a total thickness of 25.4 mm), increasing the thermal displacement compared to traditional panels [26]. The PCMs were then integrated as encapsulated material in bricks, reducing the temperature peaks by 2.5 °C and the thermal amplitude by 5 °C and increasing the thermal phase shift by 3 h [27]. Alongside these experiments, the most widespread application today is plasterboard panels, which integrate PCM Micronal DS5001X. These products integrate up to 45% of the weight of PCMs and are able to absorb five times the thermal energy of a traditional plasterboard sheet and the same energy of a full 12 cm wall [28]. One study found correlations between materials composed of gypsum and gypsum and PCM [29].

PCMs are already integrated into commercial products for the opaque envelope and technologies for the transparent envelope. For the opaque casing, there are various products in the market, including SmartBoard plasterboard panels with integrated PCMs produced by Knauf or the DuPont Energain panels produced by DuPont (5 mm thick light panels), which can be installed on interior partitions, ceilings or external walls (from the inside). As for the transparent envelope, an innovative glass facade system with PCM materials has been produced by the Swiss company GlassX. The system is defined by a multilayer façade composed of external safety glass, an air chamber with solar shading panels and 20 mm of noble gas, low-emissivity safety glass, a cavity with 20 mm of noble gas, low-emissivity safety glass, a cavity with 24 mm of phase change panels and safety glass that can be screen printed with ceramic material as needed. The product allows for diffused natural lighting with a thermal transmittance of about 0.48 W/m^2^K, a value very close to that of opaque walls, set, for example, in the Lazio region, at 0.29 W/m^2^K. Alongside these integrated systems, there are also specific products on the market based only on the use of PCMs. Among these, we can find a bag that contains PCMs and can be easily integrated into systems for false walls and false ceilings.

This article contributes to the research and studies a mix of cement and PCM with the aim of testing the mechanical and thermal performance of the compound. The paper presents the first results of a research conducted independently but which clearly refers to the studies already carried out. These studies are taken as a starting point. Compared to previous studies, this study analyzes a compound with new percentages (7%) and for both cases analyze mechanical and thermal performance in an original way. The work is structured as follows: Section 1 presents a brief state of the art to frame the topic, Section 2 contains the methodological approach, Section 3 describes the results, Section 4 deals with the discussion of results, while conclusions are drawn in Section 5.

## 2. Materials and Methods

This research, part of this general framework, has the goal of analyzing the role that smart and composite materials play in the definition of a new sustainable architecture, adapted to the challenges dictated by contemporary times and by CC.

This study aims to develop an innovative building component that combines high energy performance with reduced thickness and weight. For this reason, the potential of using PCMs in cement-based mixes is investigated, comparing the performance of a traditional mix with two innovative mixes made with the addition of 3% and 7% PCM.

The present research is divided into two main phases. In both phases, mixes with different percentages of PCM will be analyzed in order to define thermal and mechanical performance. In a first phase, whose results are presented in this paper, two different mixes respectively with 3% and 7% of PCM are analyzed. These mixes partly refer to previous studies in the scientific literature [19,20,22]. Starting with mixes already tested is useful to have comparable data.

In a second phase (next steps of the research) the study will analyse also others mixes. The PCM percentage into next mixes will be defined considering both the literature review and the results of this first phase of experimentation. For this reason, the results of these first two mixes will be used to better define the next mixes with other percentages in order to use the obtained data to better calibrate the following mixes.

The measurements, carried out in laboratories on samples of different formats, aimed to characterize the new material, analyzing its mechanical and thermal performance.

As regards the first aspect, the specimens of the PCM—cement mix were subjected to mechanical tests to evaluate the indirect tensile strength (bending test) and the compression behavior. The tests were performed following the EN 1015-11:2019 standard [30] for slender prismatic specimens. Specifically, three bending tests and six compression tests were performed for each product mix.

As regards the second aspect, the thermal conductivity far from the phase transition temperatures was measured in the laboratory, to define the behavior of the product when the PCM is in a completely solid and completely liquid phase. Subsequently, the behavior of the specimens in the temperature range that includes the solid–liquid passage of the PCM was analyzed to define the heat storage capacity of the cement mortar mixed with the phase change material.

### Mixes Used and Samples Made

The experimentation work, carried out at the Proof testing and Research in Structures and Materials (PRiSMa) Laboratory of the University of Roma Tre, tested two different mixes characterized by different percentages of PCM (3% and 5% PCM), as reported in Table 1. The choice of these mixes refers to data taken from the scientific literature [19,20,22]. In particular, in these references, the mix was made with PCM percentages of 3% and 5%. For this reason, the 3% mix has been chosen in order to have a term of comparison with studies already carried out and therefore to have comparable data, while the 7% mix, on the other hand, is due to the desire to increase the percentage more than other studies in the literature.

To develop the mixes, in particular, the products used were Class 42.5 N Portland Cement, quarry aggregates (sand) and Micronal Nextek 24D microencapsulated PCM. In this first phase of research, no additives or other components were added.

**Figure 1:**
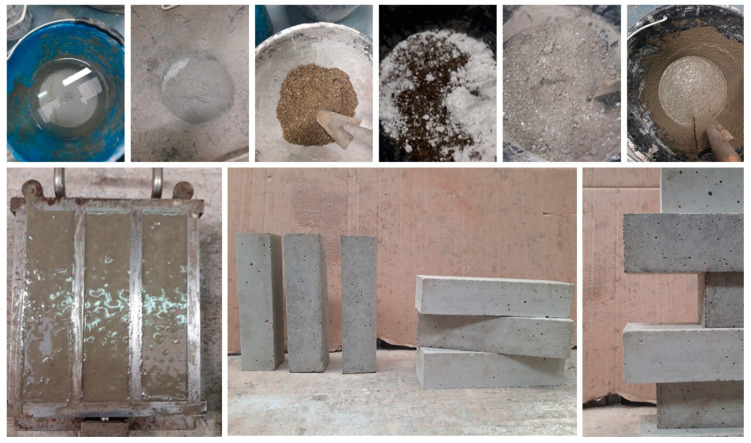
Preparation phase of the specimens (**top**), curing phase and specimens made (**bottom**).

Each mix was prepared by manual mixing, placed in special molds and left to mature for 28 days (Figure 1). For the mechanical tests, three specimens were produced for each mix with dimensions of 40 × 40 × 160 mm (Figure 4). Two specimens with dimensions of 100 × 100 × 100 mm and one with dimensions of 400 × 400 × 50 mm were produced for the thermal tests.

## 3. Results

### 3.1. Mechanical Properties

**Figure 2:**
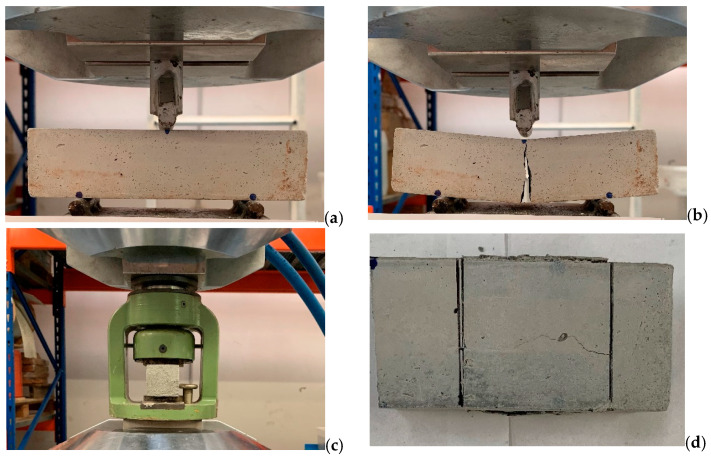
Mechanical tests on the prismatic specimen: (**a**) set-up for the bending test; (**b**) break of the specimen after the bending test; (**c**) set-up for the compression test on the half-part of the prismatic specimen; (**d**) 40 mm imprint after the compression test.

The prismatic specimens with dimensions of 40 × 40 × 160 mm were subjected to mechanical tests to evaluate their tensile and compressive behavior. The tests were performed following the EN 1015-11:2019 standard [30]. The procedure involves executing a bending test (Figure 2a) in order to evaluate the indirect tensile strength. Then, once the specimen breaks in the middle (Figure 2b), the compression test (Figure 2c) is performed on the two resulting half-parts of the prism with a 40 mm imprint (Figure 2d). Specifically, three bending tests and six compression tests were performed for each product mix. Table 2 highlights the results obtained, evaluated as the average of the values of all the tests carried out. In general, the results show how the addition of PCM reduces the mechanical performance of the mix compared to the traditional compound. Despite this reduction, good mechanical behavior can still be observed for the compressive strength of the 3% mix, while the values drop considerably for the 7% mix.

### 3.2. Thermal Properties

The HP 1-PCM 3% sample was used to assess the thermal conductivity both above (with the PCM in the solid phase), the change of state temperature and at or below the phase change temperature (with at least a partial melting of the PCM).

**Figure 3:**
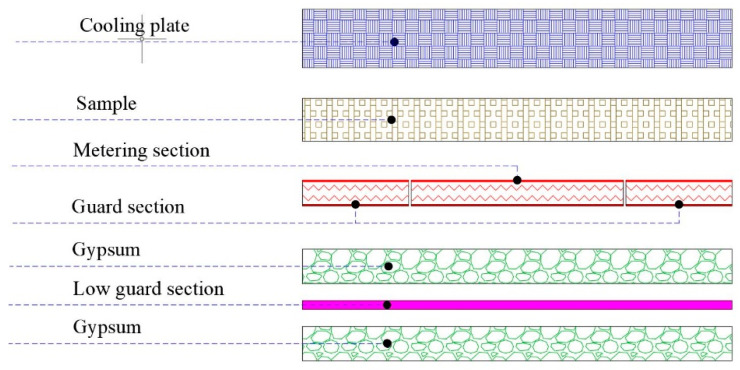
Section of the guarded hot plate apparatus.

The measurement of thermal conductivity was executed with a guarded hot plate described below (Figure 3):

- A hot plate divided into a square element of 250 × 250 mm (the measuring zone), supplied with an assigned power rate, and a frame element with a total thickness of 125 mm (the guard zone), kept at the same temperature of the previous one by a closed-loop control system, in order to avoid lateral dispersion from measuring zone. The two parts are realized in aluminium with a thickness of 30 mm and internally heated by heating cartridges;
- A cold plate (500 × 500 mm) made of a parallelepiped, constituted by a tank with an internal spiral circuit where the chilled water flows;
- A second guarded hot plate to prevent a downward heat flux placed beneath the whole surface of the hot plate and kept at the same temperature.

The control system drives the cartridge heaters in the LabView environment using a proportional–integral–derivative (PID) control. As the electric resistance of the cartridge is known, the current measurement provides the heat flux due to the Joule effect. The sample surface temperatures are determined by three sensors positioned on the two sides. The thermal conductivity is calculated with the following equation:

λ = (φ s)/(A (T_h_ − T_c_)) [W/mK] (1)

where φ is the measured thermal flux provided to the metering zone (W), s is the thickness of the sample (m), A is the area involved in the heat flow (m^2^, metering zone area), and T_h_ and T_c_ are respectively the average surface temperature (°C) on warm and cold sides of the sample. EN 12667 [26] is the standard that defines the thermal conductivity measurement with the guarded hot plate and the conditioning process of the sample before the test.

**Figure 4:**
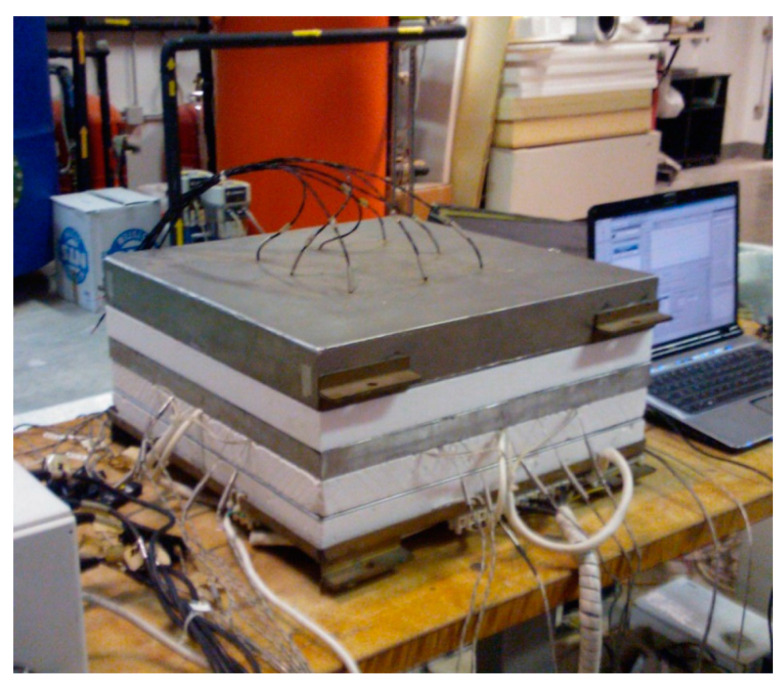
Sample during the hot-plate analysis.

The results of the thermal analysis executed on the sample (Figure 4) are reported in Table 3.

**Figure 5:**
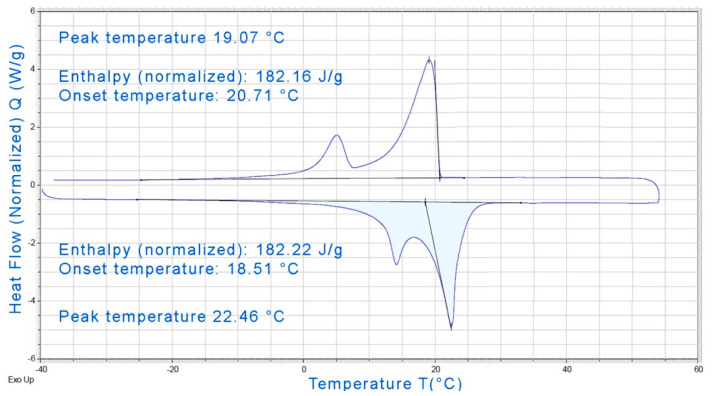
Differential scanning calorimeter test results of the PCM.

The phase change behavior is described by the differential scanning calorimeter test conducted by the PCM manufacturer (Figure 5).

## 4. Discussion

### 4.1. Mechanical Properties

The mechanical tests performed on the prismatic specimens highlight how the strength decreases as the PCM ratio increases. A rise from 3% to 7% of the PCM content implies a 50% reduction in the indirect tensile strength, while the compression is reduced to a third. However, according to the EN 998-2:2016 standard [31], both mixes may be considered suitable for masonry structures and may be classified as M5 and M15 (Table 3). Considering that the Italian Technical Standards for Buildings [32] require that the mortar must have a minimum compressive strength of 2.5 MPa to be employed for load-bearing masonries, both mixes seem to be suitable for this purpose, although a standard cementitious mortar generally has a compressive strength higher than 8.0 MPa [32]. Lower strength requirements, instead, are expected for plastering mortars (Table 4), thus the 3% mix may fall into the CS IV category, while the other mix falls into the CS III category [33].

Different considerations may be made about the employment of these mixes for the repair of concrete structures. In fact, the classification given by the EN 1504-3:2005 standard [34] requires very high strength for both structural and nonstructural use. Then, in this case, only the 3% mix may be included in the R2 class for nonstructural employment, while the 7% mix is completely excluded. Comparing the data obtained from the experimentation with those present in the literature it is possible to evaluate how, although there are some differences, it is possible to find similarities (Table 5).

In conclusion, the mechanical properties of the mixes are consistent with their employment for masonry construction both as plaster and as bedding mortar for load-bearing walls, opening up interesting research and development scenarios.

### 4.2. Thermal Properties

The mix shows good behavior in terms of insulating properties, in good agreement with the literature outcomes [19], confirming thermal conductivity values typical of lightened mixtures, as reported in [35], where the thermal conductivity values span from 0.43 to 0.56 W/mK, depending on the aggregate addition.

The hot-plate tests underline a light increase in the thermal conductivity in the temperature range where the PCM is completely solid, with respect to the zone where it is found in the liquid state, again confirming results that have already been obtained by other researchers.

The PCM calorimeter test indicates a temperature range for the phase change around 19 °C in heating and 22 °C in cooling, making it particularly suitable for building applications.

Indeed, the transition occurring around 20 °C makes the mix efficient during the hot season in moderate climates, as the daily external temperature excursion may easily include the phase change.

Therefore, the heat storage capacity of the PCM is completely exploited in a considerable fraction of the year.

## 5. Conclusions

The evidence of climate change and the serious consequences associated with it require a radical rethinking of urban settlements, buildings and construction materials. In this context, the need to develop increasingly sustainable buildings also appears clear through smart materials with high performance and reduced carbon emissions.

This research work is part of this general framework and aims to develop an innovative smart material, consisting of a mix of cement, aggregates (sand) and PCM. It is precisely the PCMs integrated into the mix that allow improving its thermal performance and, with an increase in performance, it can correspond to a reduction in the quantity of other materials (sand and cement), thus allowing the development of a product with high performance and reduced carbon emissions.

A synthetic state of the art is presented in the first part. On this basis, samples were developed featuring two mixes with different amounts of PCM (3–7%). Then, mechanical and thermal characterization was developed.

From the analysis of the mechanical performance, it emerges that the contribution of the PCM reduces the mechanical strength of the compound, as expected and already found in the literature. In particular, the mechanical tests performed on the prismatic specimens highlight how the strength decreases as the PCM ratio increases. A rise from 3% to 7% of the PCM content implies a 50% reduction of the indirect tensile strength, while the compression is reduced to a third. However, both mixes may be considered suitable for masonry structures and may be classified as M5 and M15. These results are aligned even if different with respect to some scientific literature data. Indeed, even if the absolute values appear slightly different between the data found in the literature and those obtained from the experimentation, the percentage of reduction in mechanical performance appears similar, with a reduction of 5o% and 30% for medium compressive strength and medium indirect tensile strength respectively.

From the analysis of the thermal performance, it emerges that the mix presents good behavior in terms of insulating properties, in good agreement with literature outcomes. The hot-plate tests underline a light increase of the thermal conductivity in the temperature range where the PCM is completely solid, with respect to the zone where it is found in the liquid state. The test indicates a temperature range for the phase change around 19 °C in heating and 22 °C in cooling, making it particularly suitable for building applications.

In conclusion, considering the mechanical properties of the mixes, it is possible to observe how the mixes are consistent with their employment for masonry construction both as plaster and as bedding mortar for load-bearing walls, opening up interesting research and development scenarios. On the other hand, considering the thermal properties of the mixes, it is possible to observe good behavior in terms of insulating properties. The results highlight the potential of composite materials in terms of increasing the performance and applicability to the construction sector. In this sense, the next phases of the research aim at engaging new mixes with different percentages and with the inclusion of recycled materials.

## Figures and Tables

**Table 1:** Mixes adopted, highlighting the weight and percentage of the final compound.

Structure Sample Mix				
Mix	HP 1-PCM 3%		HP 1-PCM 7%	
	Grams	%	Grams	%
PCM	90	2.8%	260	6.9%
Water	400	12.5%	600	16.0%
Cement	600	18.8%	700	18.6%
Sand	2100	65.8%	2200	58.5%
TOT	3190	100.0%	3760	100.0%

**Table 2:** Results of the mechanical tests for the two mixes.

Mix	Medium Indirect Tensile Strength	Medium Compressive Strength
%	f_bm_% (MPa)	f_cm_% (MPa)
3%	4.06	15.76
7%	2.08	5.87

**Table 3:** Thermal conductivity measurements of the HP 1-PCM 3% sample at different temperatures.

High-temperature test (PCM in solid phase)	T_lab_	23	°C
	R.H._lab_	40	%
	Surface temperature, hot side	28.4	°C
	Surface temperature, cold side	20.0	°C
	Average temperature of the sample	24.2	°C
	Thermal flux supplied to the metering zone	5.50	W
	Thermal conductivity	0.44	W/mK
	Uncertainty	1.0	%
Low-temperature test (PCM in liquid phase)	T_lab_	23	°C
	R.H._lab_	45	%
	Surface temperature, hot side	21.6	°C
	Surface temperature, cold side	14.5	°C
	Average temperature of the sample	18.1	°C
	Thermal flux supplied to the metering zone	3.87	W
	Thermal conductivity	0.37	W/mK
	Uncertainty	1.0	%

**Table 4:** Comparative table between data in the literature and data obtained with experimentation, with medium indirect tensile strength (1) and medium compressive strength (2).

Standard	Typologies	Category	N/mm^2^
EN 998-1: 2016	Plastering mortar	CS I	0.4–2.5
		CS II	1.5–5.0
		CS III	3.5–7.5
		CS IV	≥6
EN 998-2: 2016	Mortar for masonry	M1	1
		M2.5	2.5
		M5	5
		M10	10
		M15	15
		M20	20
EN 1504-3: 2005	Mortar for nonstructural repair	R1	≥10
		R2	≥15
	Mortar for structural repair	R3	≥25
		R4	≥45

**Table 5:** Comparative table between data in the literature and data obtained with experimentation, with medium indirect tensile strength (1) and medium compressive strength (2).

Reference		0% PCM	3% PCM	5% PCM	7% PCM	Performance Reduction (%)
[19]	(1)	6	4			−33%
	(2)	47	28			−41%
[22]	(1)	8.55		6		−30%
	(2)	64.4		37		−43%
Experimental data	(1)	[19]	4.06		2.08	−33% −66%
	(2)		15.76		5.87	−70% −88%

## Data Availability

Not applicable.
